# Nanocrystalline diamond protects Zr cladding surface against oxygen and hydrogen uptake: Nuclear fuel durability enhancement

**DOI:** 10.1038/s41598-017-06923-4

**Published:** 2017-07-25

**Authors:** Jan Škarohlíd, Petr Ashcheulov, Radek Škoda, Andrew Taylor, Radim Čtvrtlík, Jan Tomáštík, František Fendrych, Jaromír Kopeček, Vladimír Cháb, Stanislav Cichoň, Petr Sajdl, Jan Macák, Peng Xu, Jonna M. Partezana, Jan Lorinčík, Jana Prehradná, Martin Steinbrück, Irena Kratochvílová

**Affiliations:** 1 Czech Technical University in Prague, Faculty of Mechanical Engineering, Technická 4, Prague 6, CZ-160 07 Czech Republic; 2 Institute of Physics of the Czech Academy of Sciences, Na Slovance 2, CZ-182 21 Prague 8, Czech Republic; 3 RCPTM, Joint Laboratory of Optics of Palacký University in Olomouc and Institute of Physics of the Czech Academy of Sciences, 17. listopadu 12, CZ-771 46 Olomouc, Czech Republic; 4 University of Chemistry and Technology, Power Engineering Department, Technická 3, Prague 6, CZ-166 28 Czech Republic; 5 Nuclear Fuel Division, Westinghouse Electric Company, 5801 Bluff Road, Hopkins, SC 29209 USA; 6 Westinghouse Churchill Site, 1332 Beulah Rd., Pittsburgh, PA 15235 USA; 7 Research Centre Řež, Hlavní 130, CZ-250 68 Husinec-Řež, Czech Republic; 8 Institute for Applied Materials (IAM), Karlsruhe Institute of Technology, Hermann-von-Helmholtz-Platz 1, 76344 Eggenstein-Leopoldshafen, Germany

## Abstract

In this work, we demonstrate and describe an effective method of protecting zirconium fuel cladding against oxygen and hydrogen uptake at both accident and working temperatures in water-cooled nuclear reactor environments. Zr alloy samples were coated with nanocrystalline diamond (NCD) layers of different thicknesses, grown in a microwave plasma chemical vapor deposition apparatus. In addition to showing that such an NCD layer prevents the Zr alloy from directly interacting with water, we show that carbon released from the NCD film enters the underlying Zr material and changes its properties, such that uptake of oxygen and hydrogen is significantly decreased. After 100–170 days of exposure to hot water at 360 °C, the oxidation of the NCD-coated Zr plates was typically decreased by 40%. Protective NCD layers may prolong the lifetime of nuclear cladding and consequently enhance nuclear fuel burnup. NCD may also serve as a passive element for nuclear safety. NCD-coated ZIRLO claddings have been selected as a candidate for Accident Tolerant Fuel in commercially operated reactors in 2020.

## Introduction

To satisfy the demands for safe operation and to extend the lifetime of nuclear fuel, it is necessary to decrease the surface corrosion of zirconium fuel claddings. Zirconium alloys are standard structural materials used in the cores of water-cooled nuclear power reactors^1–5^. A significant problem is posed by the dissociation of water molecules at the surface of a zirconium alloy, even at standard operating temperatures, thus allowing both oxygen and hydrogen to penetrate into the alloy. As the alloy oxidizes and zirconium hydrides form, the ductility of the nuclear rod decreases. Oxygen and hydrogen uptake into the Zr alloy changes the mechanical properties of the core components, which are important criteria for the licensing of nuclear fuel^4–7^. Thus, the protection of the surfaces of zirconium fuel rods against corrosion in nuclear reactor environments may significantly prolong nuclear fuel usage^8–14^. Recently, many typically homogeneous materials have been applied to protect the surfaces of zirconium alloys against direct interaction with water molecules, but the majority of these efforts have not met with significant success^5–7^.

In this work, we demonstrate and describe a novel means of protecting ZIRLO surfaces against O and H uptake at both accident and working temperatures in water-cooled nuclear reactor environments. Tubular ZIRLO fuel claddings (tubes) and ZIRLO plates were protected by thin (300–700 nm) composite nanocrystalline diamond (NCD) layers deposited onto the ZIRLO surfaces with a microwave plasma-enhanced linear antenna chemical vapor deposition (MW-LA-PECVD) apparatus^15–18^ (Fig. S1). NCD layers have a high thermal conductivity, provide environmental stability and exhibit low chemical reactivity^16, 17^. As we have previously shown^15–17^, NCD layers are able to adhere to Zr alloy surfaces at high temperatures and possess suitable thermal expansion properties.

The behavior of NCD coated Zr alloy in a neutron flux environment was simulated by Fe ion beam irradiation. After Fe^2+^ beam irradiation (10 displacements per atom/dpa, 3 MeV Fe^2+^, fluency of 1.95 × 10^16^ cm^−2^), and hot steam processing NCD films grown in the MW-LA-PECVD apparatus have been shown to exhibit structural integrity^16^. The diamond radiation damage dose dependence was shown in refs 19 and 20. In ref. 19 the effects of neutron irradiation ((1–5).10^20^ cm^−2^ fluence range) on chemical vapor deposited diamond were investigated. Despite an increase in unit-cell volume (4.5%) the crystalline structure remain stable^19^. In the case of X-ray study of neutron irradiated diamonds^20^ (4.8 and 16.3 × 10^20^ cm^−2^ fluence) the irradiated diamond density decrease (accompanied by structural changes) was in majority cases between 12 and 25%, with the average of 17%.

The NCD grown in the MW-LA-PECVD apparatus is a composite material: it consists of diamond grains (sp^3^-hybridized phase, >96%) and graphite (sp^2^ phase)^15^. Such NCD layers with thicknesses from 300 nm to 700 nm have been found to significantly decrease oxygen and hydrogen uptake into ZIRLO fuel tubes and plates processed in Westinghouse Electric Company facilities, in accordance with ASTM standard procedures (360 °C, hot water exposure for 6 days up to 195 days)^21^. In addition, we evaluated NCD’s protective performance for 4 days in hot steam at 400 °C (15 MPa, less than 10 ppb of oxygen) and at extremely high steam temperatures of 900–1100 °C for 1 hour and 1200 °C for 30 min.

We extensively studied and interpreted the detailed role of NCD layers in preventing the penetration of oxygen and hydrogen into ZIRLO surfaces and hindering Zr oxidation, by using several different characterization methods: X-ray photoelectron spectroscopy (XPS), Raman spectroscopy, scanning electron microscopy (SEM), energy-dispersive spectroscopy (EDS), thermogravimetry, mass spectrometry, secondary ion mass spectrometry (SIMS), capacitance measurements and NanoESCA. The relevance of each of these techniques with respect to the subject of this research are discussed in refs 22–32. The adhesion of the protective NCD layers on the nuclear fuel cladding surfaces before and after oxidation due to exposure to hot steam and water was investigated through analysis of the mechanical and tribological properties of the samples. This analysis was conducted on the basis of the approaches presented in refs 31–34. We demonstrated that because of the specific composition of the NCD, carbon atoms from an NCD layer enter the underlying Zr material and significantly modify its structural, chemical and physical parameters in a favorable fashion. These changes result in lower uptake of hydrogen and oxygen into the ZIRLO, thereby leading to lower degradation of fuel tubes and plates in hot steam or water environments.

In this paper, we show that at high temperatures (up to 1200 °C), NCD layers can fully serve as passive elements for nuclear safety. NCD layers provide crucial and effective protection against Zr alloy oxidation and hydration under standard reactor operating conditions (360 °C, hot water, ASTM standard procedures)^21^. Such protective NCD layers can prolong the lifetime of nuclear cladding and, consequently, enhance nuclear fuel burnup. However, the main novelty and relevance of this work lie in providing a fundamental explanation of the causes of the astonishingly high corrosion prevention of Zr alloys, which is achieved through the deposition of composite NCD layers. Despite long-term exposure to a corrosive environment and specific nature of the heterogeneous NCD material (diamond and graphitic phases), the anti-corrosive effect of such protective NCD layers is very significant. On the basis of a wide range of analyses, we describe in detail the complex and specific mechanism by which heterogeneous NCD layers protect ZIRLO surfaces against corrosion.

## Results

### Weight gain of samples after hot steam or hot water processing

**Figure 1:**
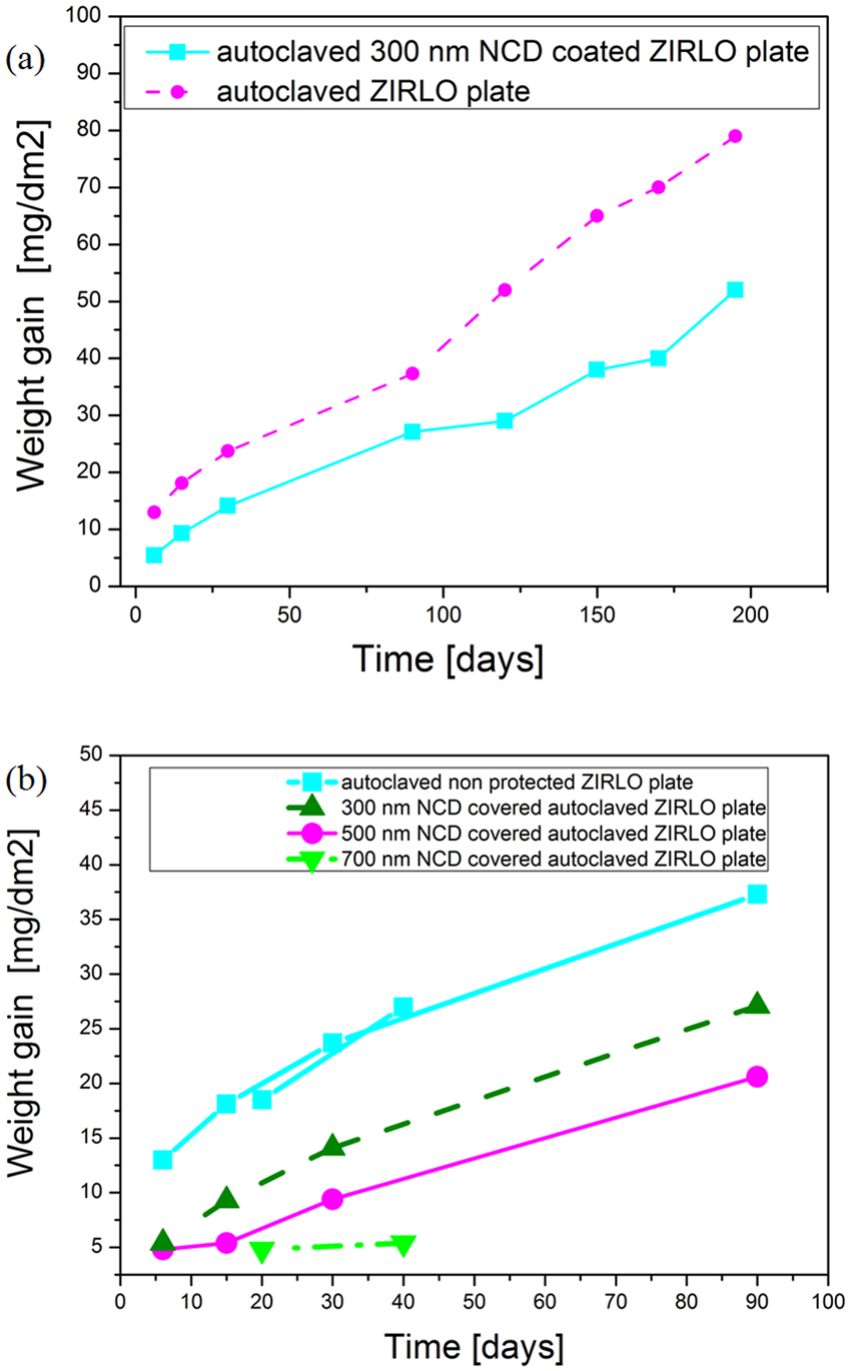
(**a**) weight gains (mg/dm^−2^) measured after 6, 15, 30, 90, 120, 150, 170 and 195 days of exposure to hot water (360 °C) for uncoated and 300 nm NCD-coated ZIRLO plate samples. (**b**) weight gains (mg/dm^−2^) measured after 6, 15, 20, 30, 40 and 90 days of exposure to hot water (360 °C) for uncoated, 300 nm NCD-coated, 500 nm NCD-coated and 700 nm NCD-coated ZIRLO plate samples. Thicker NCD layers provide ZIRLO plates with stronger protection against oxidation than thinner layers do.

To determine the protective capabilities of NCD layers under standard reactor conditions, ZIRLO samples (tubes and plates) coated with 300 nm, 500 nm and 700 nm NCD layers were processed in hot water at 360 °C for durations ranging from 6 days up to 195 days (processed in a pressurized water reactor (PWR), in accordance with ASTM standard procedures)^21^. After these tests, the ZIRLO samples were found to still be coated with their protective NCD layers (confirmed through Raman spectroscopy and SEM imaging, as shown below). Table 1 and Fig. 1 display the weight gains of standard and NCD-protected ZIRLO tubes and plates after processing in hot water (360 °C). The weight gain was determined as the difference in the sample weight before and after oxidation, normalized to the total exposed area of the sample surface. According to^12^, these weight gains are caused by oxidation and hydrogen uptake into the samples. The weight gains were measured after the hot water processing (360 °C) of ZIRLO samples with NCD coatings (300 nm, 500 nm or 700 nm) and uncoated ZIRLO samples. For all of the hot-water-processed samples, the NCD-coated Zr samples showed lower weight gains compared with those of the uncoated samples (Table 1 and Fig. 1). Similar results were observed after 4 days of exposure to hot steam (400 °C): the weight gain for a ZIRLO sample coated with a 300 nm NCD layer was 7.8 mg/dm^2^, whereas the weight gain for an uncoated ZIRLO sample was 13.2 mg/dm^2^. All weight gains were subject to an uncertainty (experimental plus numbers rounding) of less than 2%.

**Table 1:** Weight gains (mg/dm^2^) measured after autoclave tests of uncoated ZIRLO samples (tubes and plates) and samples coated with 300 or 500 nm of NCD.

Sample type	NCD layer thickness (nm)	Weight gain (mg/dm^2^) of autoclaved samples							
		6 days	15 days	30 days	90 days	120 days	150 days	170 days	195 days
Tube	300	9.4	15.5	19.6	33.3	31	30	41	—
Tube	500	7.7	13.6	19.1	31.5	—	—	—	—
Plate	300	5.4	9.3	14.1	27.1	29	38	40	52
Plate	500	4.8	5.4	9.4	20.6	—	—	—	—
Tube	0	15.1	20.6	26.2	40.3	52	66	68	—
Plate	0	13.0	18.1	23.7	37.3	52	65	70	79

The samples were processed in 360 °C hot water for 6, 15, 30, 90, 120, 150, 170, and 195 days under primary circuit conditions in a PWR in accordance with ASTM standard procedures^21^. The greater relative weight gain of the uncoated ZIRLO plates and tubes indicated stronger oxidation of the unprotected surfaces.

Table 1 and Fig. 1 show that in the case of the thickest (700 nm) NCD coatings, the smallest oxidation (weight gain) of the protected ZIRLO was measured. For ZIRLO plates coated with 700 nm NCD layers, the surface oxidation/weight gain was decreased by more than 50% compared with that of the non-protected ZIRLO plates. The weight gains of ZIRLO plates coated with 700 nm NCD and uncoated ZIRLO plates after exposure to 360 °C hot water for 20 days were 4.8 mg/dm^2^ and 18 mg/dm^2^, respectively. When the exposure was prolonged to 40 days, the same trend was observed: the weight gain was 5.4 mg/dm^2^ for ZIRLO plates coated with 700 nm NCD and 31 mg/dm^2^ for uncoated ZIRLO plates. From Table 1 and Fig. 1, a systematic decrease in weight gain was evident for ZIRLO plates coated with 300 nm, 500 nm and 700 nm of NCD.

In the case of ZIRLO plates that were mounted vertically in the deposition chamber, all surfaces were covered with more homogeneous NCD layers compared with those coating the ZIRLO tubes^15^. This observation was because the inner surfaces of the ZIRLO tubes were not fully covered by the NCD (as confirmed by SEM imaging and Raman spectroscopy), as the plasma was unable to fully penetrate into a tube’s internal structure. Therefore, an apparent decrease in corrosion resistance was observed for the ZIRLO tubes. These results demonstrate the importance of coating homogeneity. For this reason, the results obtained for the NCD-coated ZIRLO plates are more representative (Table 1 and Fig. 1). In practice, during NCD growth in the MW-LA-PECVD apparatus, both ends of each tube could be closed with metallic closures to prevent the inner part of the tube from being coated with an NCD layer.

For the samples processed in hot water (360 °C) for 90 days, the hydrogen concentrations were measured by means of mass spectrometry. The obtained results showed that uncoated ZIRLO tubes had a higher hydrogen concentration (37 ppm) compared with 500 nm NCD-coated ZIRLO tubes (31 ppm) and plates (21 ppm) (Table S1). The same analysis was conducted for uncoated and 300 nm NCD-coated ZIRLO tubes after hot steam processing (1100 °C for 60 min and 1200 °C for 20 min) (Table S2). The highest hydrogen concentration was found in the uncoated samples (Table S1). For hot steam processing at temperatures above 900 °C, the uptake of hydrogen into the unprotected material was very high (Table S3). Compared with ZIRLO samples protected with 500 nm of NCD, the hydrogen concentration in the unprotected ZIRLO samples was found to be larger by one order of magnitude (Table S3). The relative standard deviation of the hydrogen concentrations was less than 6.4%. The precision of all hydrogen concentration measurements was 0.5% (mass spectrometer model: G8 GALILEO).

### Mechanical and tribological measurements

NCD layers exhibit stable and strong adhesion to ZIRLO surfaces, protecting them from direct interaction with water. The improvement in the scratch resistance of a ZIRLO plate after the application of an NCD coating (300 nm thick) is shown in Fig. S2, in which on-load depth measurements are presented for uncoated and NCD-coated ZIRLO before and after hot steam processing (400 °C, 4 days). Because of the higher hardness of the NCD (approx. 18.5 GPa), a low depth profile was clearly seen for the NCD-coated ZIRLO substrate. ZIRLO, by contrast, is a softer material with a lower scratch resistance. The formation of zirconium oxide on the surface of ZIRLO during exposure to hot steam improved its scratch resistance. However, the adhesion of this oxide layer was poor, thus leading to sudden ZrO_2_ failure and delamination, exposing the underlying ZIRLO; note the sudden drop in depth at a scan distance of ~330 µm. The critical load was approximately 370 mN. In the case of the NCD-coated ZIRLO, there were no signs of such failure after hot steam exposure. Analysis of the residual wear track also revealed a lower level of cracking. Similar results were obtained for ZIRLO tubes coated with 700 nm thick NCD films. Notably, a 500 nm thick NCD layer was found to provide less protection than a 700 nm layer.

The deposition of the 300–700 nm thick NCD layers improved the mechanical stability and overall integrity of the ZIRLO during and after hot water/steam exposure and provided an extra level of mechanical durability. In summary, the scratch resistivity of NCD-protected ZIRLO remained unchanged after 4 days of exposure to hot steam at 400 °C.

### Thermogravimetry, mass spectrometry and optical microscopy of the metallographic cross sections of samples

Thermogravimetry experiments were performed to measure the mass changes occurring during high-temperature steam oxidation. Furthermore, off-gas analysis was performed to measure hydrogen production (Table S1). After exposure for 60 min at 900 °C, 1000 °C, or 1100 °C in hot steam, the 300 nm NCD-coated ZIRLO samples were still covered with an NCD layer (as confirmed by Raman spectroscopy and SEM imaging). The weight gains were measured after the hot steam tests for the NCD-coated and unprotected ZIRLO samples. After hot steam exposure, higher relative weight gain (surface oxidation) and hydrogen production were measured for the uncoated ZIRLO samples compared with the NCD-coated ZIRLO samples. After exposure at 1100 °C, the amount of hydrogen in the uncoated samples was approximately 571 ppm, whereas for the NCD-coated samples, this value was decreased to 51 ppm (Table S3). The oxygen concentration in the outgoing gas/high-temperature steam (above 900 °C) was less than 0.08%.

**Figure 2:**
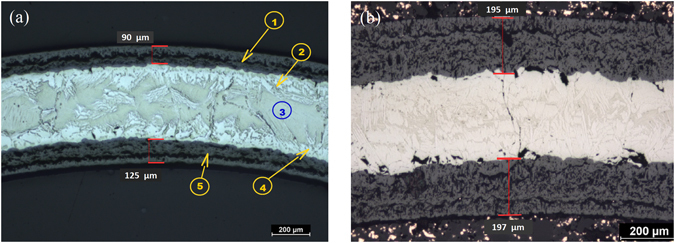
Optical microscopy images of the metallographic cross sections. (**a**) a 300 nm NCD-coated ZIRLO tube sample processed at 1000 °C in hot steam. (1) Outer-surface zirconium dioxide layer, with a thickness of 90.8 μm. (2) Outer-surface oxygen-stabilized zirconium α phase (solid Zr-O solution). (3) Initial Zr β phase (metastable modified hexagonal close-packed structure). (4) Inner-surface oxygen-stabilized Zr α phase. (5) Inner-surface ZrO_2_ layer, with a thickness of 125.02 μm. (**b**) an uncoated ZIRLO tube sample subjected to hot steam (1000 °C/1 h). On the outer surface of the tube, the ZrO_2_ thickness was 195.13 μm; on the inner surface, the ZrO_2_ thickness was 196.8 μm.

Metallographic cross sections (Fig. 2) of NCD-coated (300 nm) and uncoated ZIRLO tubes after exposure to hot steam (1000 °C) for 1 h were obtained with an optical microscope. Metallographic cross-section analysis allowed oxides and different zirconium phases to be distinguished. As discussed earlier, the inner parts of the tubes were not as effectively coated with NCD compared with the outer surfaces. For this reason, a thinner layer of ZrO_2_ was measured on the outer surface of the tube depicted in the figure (90.80 μm) compared with the inner surface (125.02 μm). However, both of these values were significantly less than those for an uncoated ZIRLO tube (195.13 μm on the outer surface and 196.8 μm on the inner surface) exposed to the same conditions. These values confirmed the protective effect of the NCD layers.

### X-ray photoelectron spectroscopy

The protective capabilities of the NCD layers were further evidenced by XPS data acquired from the NCD-coated (300 nm) and uncoated ZIRLO tubes before and after hot steam tests (in an autoclave for 4 days at 400 °C). The XPS spectra were obtained from cross sections of the samples. The results of the XPS analysis can be divided into two parts: the results of a quantitative analysis performed on the surface of each cross section at the metal-to-oxide interface and the results of an analysis of the states of carbon identified to be present in the metal and oxide layers.

Regarding the analysis of the carbon states, the presence of carbides and forms of carbon with a binding energy near 285 eV was determined from the detailed spectra of the C1s peak. The Zr 3d5/2 line of metallic zirconium (179 eV) was used as the calibration standard because of the low level of surface charging.

The presence of carbide in the ZrO_2_ layer was identified from the existence of a line with a binding energy of 281.6 eV. The difference in the carbon binding energy peaks for autoclaved NCD-coated ZIRLO compared with autoclaved uncoated ZIRLO was 0.8 eV, which was reproducibly observed in many spectra. At least two carbon states (with different binding energies) were identified in the autoclaved NCD-coated ZIRLO, as indicated by the structure of the main part of the C1s line.

**Figure 3:**
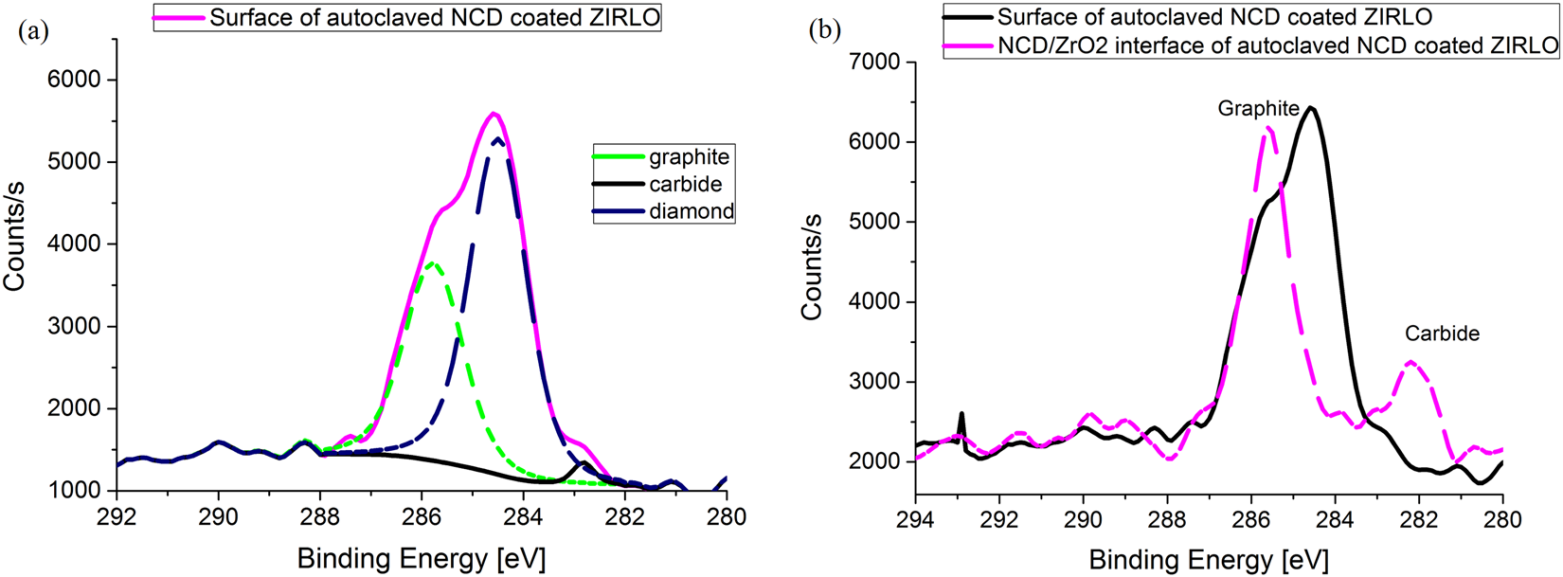
C1s lines of ZIRLO coated with NCD after calibration on the basis of the Zr 3d5/2 line. The areas analyzed were on the NCD surface and at the NCD/ZrO_2_ interface after hot steam exposure (400 °C, 4 days). Four different states of carbon are evident (**a**): graphite, with a binding energy of 285.4 eV; diamond, with a binding energy of 284.2 eV; carbon in a single bond with oxygen, with a binding energy of 286.1 eV; and carbide, with a binding energy of 282 eV. Carbides were also present below the NCD layer (**b**).

In total, four states of carbon were identified: graphite (Fig. 3), with a binding energy of 285.4 eV; diamond, with a binding energy of 284.2 eV; carbon in a single bond with oxygen, with a binding energy of 286.1 eV; and carbide, with a binding energy of 282 eV. The binding energies of graphite and diamond are relatively close to each other. These two carbon states were resolved on the basis of a set of spectra measured at several different locations in the samples. The difference in the positions of the two lines may also be the result of electrical charging, which might occur slightly differently for the two carbon forms.

The surface layer of an autoclaved uncoated ZIRLO tube was found to be chemically composed mainly of ZrO_2_, thus suggesting a large uptake of oxygen. Furthermore, after autoclaving, the amount of oxygen at the surface for uncoated ZIRLO (25%) was higher than that for the NCD-coated ZIRLO samples (14%). The uncoated autoclaved samples also contained a larger number (compared with the NCD-protected autoclaved samples) of oxygens in non-stoichiometric positions – i.e., during hot steam processing (4 days, 400 °C), oxygen defects formed in the surface layer of the uncoated ZIRLO in higher numbers than in the NCD-coated ZIRLO samples.

After the autoclaving of the NCD-coated ZIRLO samples, carbides were detected in the ZrO_2_ layer and in the Zr alloy (i.e., beneath the oxide layer). In conclusion, after the autoclave tests, the Zr surfaces of the NCD-coated ZIRLO samples were significantly affected by the presence of the NCD layer, and the effects manifested as a higher concentration of carbides and a lower concentration of oxygen. Carbides were also found in the underlying Zr alloy.

### Raman spectroscopy, scanning electron microscopy, and energy-dispersive spectroscopy

**Figure 4:**
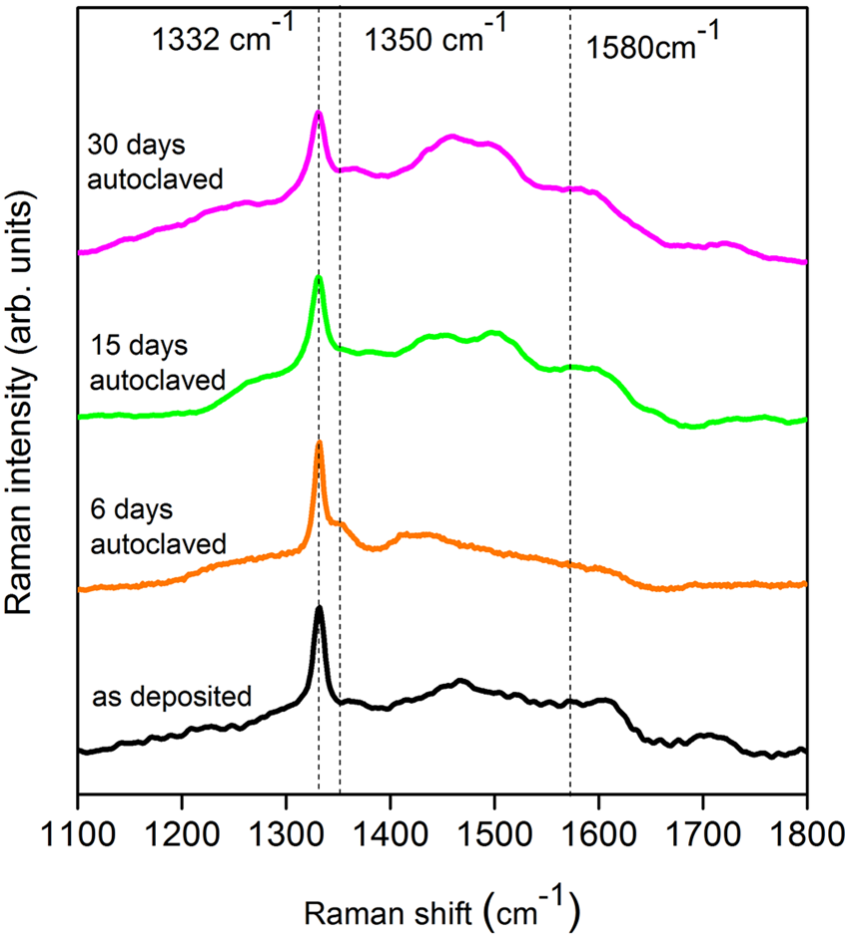
Measured 488 nm Raman spectra of 300 nm thick NCD layers on ZIRLO tubes, as deposited and after 6, 15 and 30 days of exposure to hot water at 360 °C. The 1332 cm^−1^ peak is typical of diamond. The broad band visible at 1400–1550 cm^−1^ is a signature of amorphous carbon located within the grain boundaries, the band at ~1580 cm^−1^ was assigned to graphite, and the ~1620 cm^−1^ peak was attributed to the presence of “graphite-like” disordered carbon.

The Raman spectra of the surfaces of NCD-coated ZIRLO tubes before and after exposure to hot water (360 °C) for 6 days, 15 days and 30 days are shown in Fig. 4. The spectrum of the as-deposited NCD layer showed a sharp 1332 cm^−1^ peak originating from diamond along with peaks attributed to non-diamond phases. The broad band visible at 1400–1550 cm^−1^ is a signature of amorphous carbon located within the grain boundaries, the band visible at ~1580 cm^−1^ was assigned to graphite, and the ~1620 cm^−1^ peak was attributed to the presence of “graphite-like” disordered carbon. After 6 days of autoclave exposure to hot water (360 °C), the 1350 cm^−1^ peak became visible in the spectrum, thus indicating the partial transformation of graphite into a disordered phase. With the further increase of the hot water exposure time (to 15 and 30 days), the Raman spectra of the NCD layers showed an increase in intensity and a broadening of the amorphous-carbon-related band (1400–1550 cm^−1^) and of the graphite and disordered graphite (1350 cm^−1^) peaks; these observations indicated an increasing amount of “disorganized” carbon in the layers along with a partial transformation of sp^3^ carbon into the sp^2^ phase^17, 18, 35^. The Raman spectroscopy results also confirmed that the NCD layers on ZIRLO withstood exposure to hot steam (900–1100 °C) for 1 h without exhibiting significant changes.

**Figure 5:**
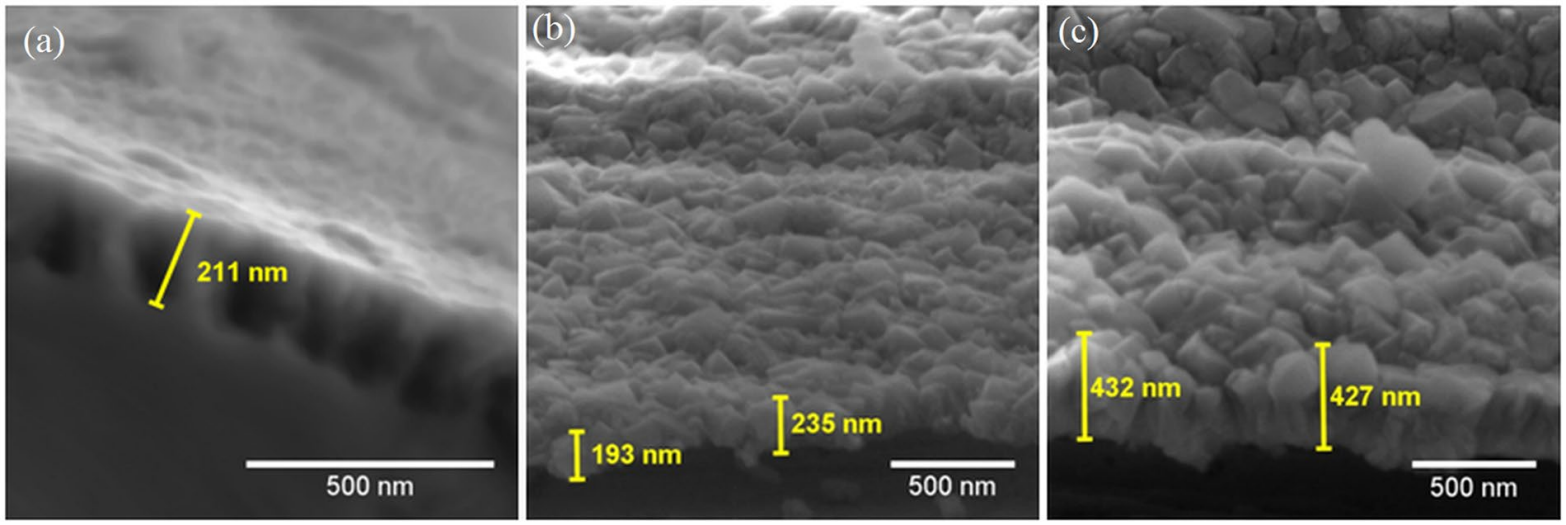
Secondary electron micrographs of NCD layer. (**a**) Thickness of 300 nm before 30 days of exposure to 360 °C hot water. (**b**) Thickness of 300 nm after 30 days of exposure to 360 °C hot water. (**c**) Thickness of 500 nm after 15 days of exposure to 360 °C hot water. No important change in the NCD layer thickness was detected, thus confirming the high durability of the layer.

**Figure 6:**
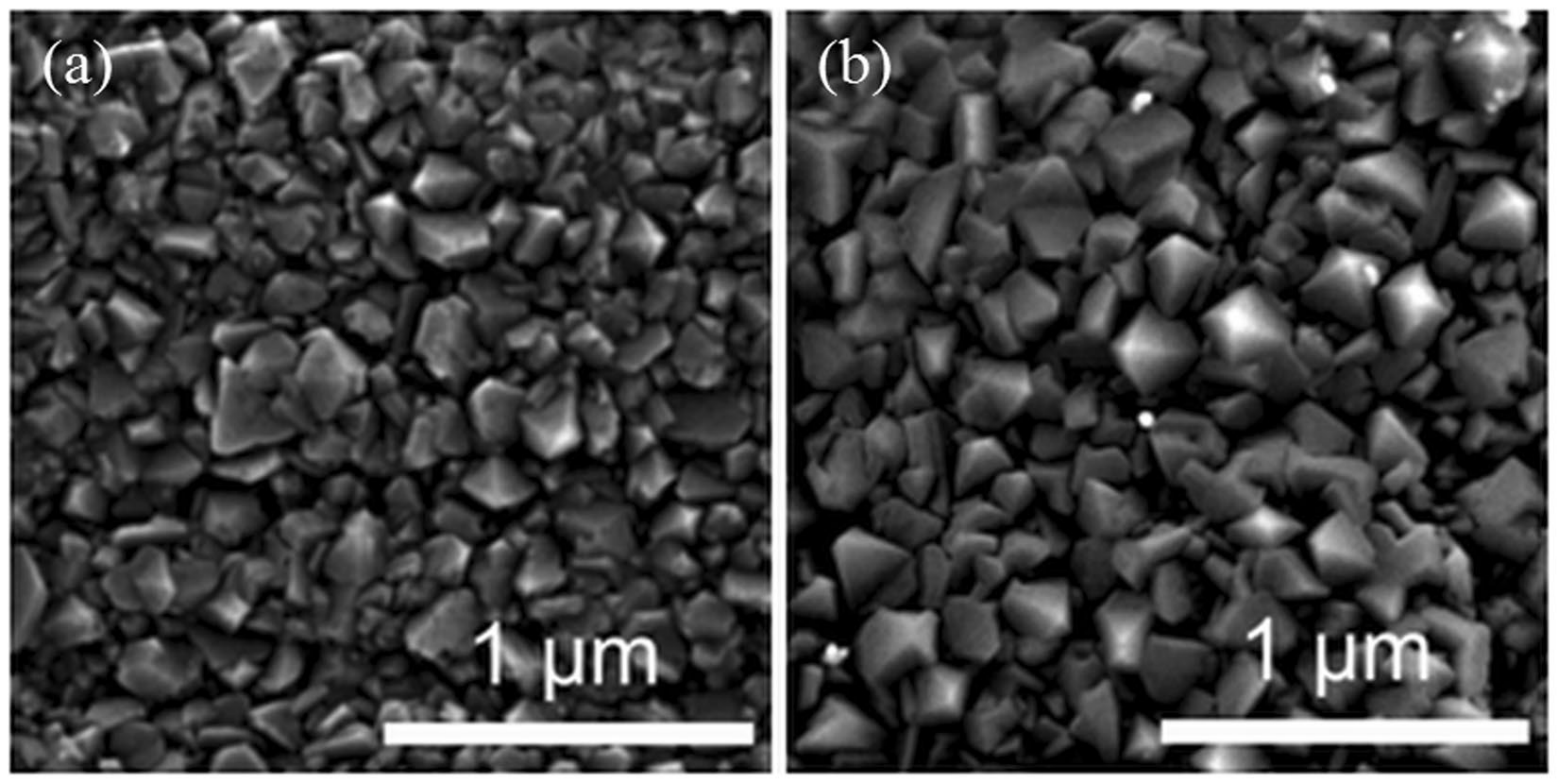
Secondary electron micrographs of the initial surface of a Zr alloy sample covered with 300 nm thick NCD (left) and the surface after 30 days of exposure to 360 °C hot water (right). No microstructural changes were observed in the hot-water-processed NCD film.

SEM revealed no important changes in the thicknesses of NCD layers subjected to hot water (360 °C) autoclaving (Fig. 5). Moreover, no microstructural changes were found in 300 nm NCD films processed in hot water for 30 days (Fig. 6).

The compositional effects of processing in hot steam (4 days at 400 °C) were studied by means of EDS in focused ion beam (FIB)-milled trenches. In the case of unprotected ZIRLO, the surface contained 18 ± 1 at.% O, the oxidized ZrO_2_ layer contained 33 ± 2 at.% O, and the bulk alloy contained 6.4 ± 0.5 at.% O. A sample protected with a 300 nm NCD layer contained 15 ± 1 at.% O at the surface, 19 ± 1 at.% O in the oxidized ZrO_2_ layer, and 6.2 ± 0.5 at.% O in the bulk alloy (Table S4). The thickness of the oxidized zone in the unprotected ZIRLO sample after treatment was 2.16 ± 0.05 μm. For the NCD-coated ZIRLO, this thickness was decreased to 1.7 ± 0.05 μm.

These results showed that the NCD layer suppressed the formation of the oxide layer after exposure to hot steam; i.e., the oxidized layer grew more rapidly on unprotected ZIRLO.

In addition, Raman spectroscopy and SEM confirmed that the outer surfaces of tube samples were more effectively coated with high-quality 300 nm NCD layers (thicker and more homogeneous NCD films and a larger relative amount of sp^3^-hybridized C), as compared with the inner surfaces of the tubes (an estimated 40% of the inner surface was covered with a thinner NCD film of only 80 to 120 nm in thickness) (Fig. S3).

### Secondary ion mass spectrometry

**Figure 7:**
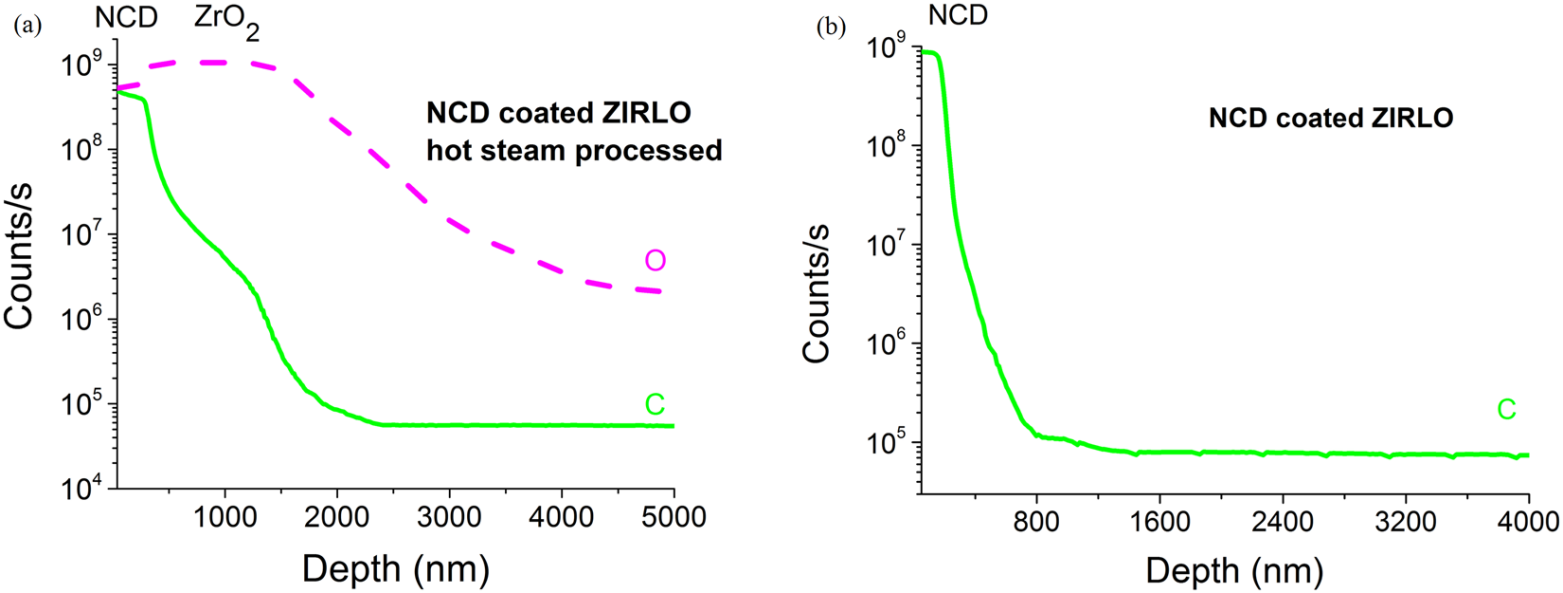
SIMS data showing that after 4 days of hot steam exposure at 400 °C, the ZrO_2_ layer in 300 nm NCD-coated ZIRLO contained C to a depth bigger than 1.5 µm (**a**), whereas in non-exposed NCD-coated ZIRLO, the majority of the carbon was contained only within the thin (0.4 µm) NCD layer (**b**). The depth profiles of C and O are in units of counts/s and labelled in the graphs.

SIMS is notable among chemical-analytical methods in that it combines high sensitivity and distinguishing capabilities with the capacity to analyze all elements and isotopes in the periodic table^23^. We used SIMS to determine the changes in the C depth profile of a 300 nm NCD-coated ZIRLO tube before and after 4 days of exposure to 400 °C hot steam. The SIMS data showed that after 4 days at 400 °C in hot steam, a large amount of C was contained in the ZrO_2_ layer formed beneath the protective NCD layer. The exposure to hot steam clearly resulted in the diffusion of carbon into the ZrO_2_ layer to a depth larger than 1.5 µm (Fig. 7a). In a reference NCD-coated ZIRLO sample (not exposed to hot steam), the majority of the carbon atoms were contained within the thickness of the NCD layer itself (Fig. 7b). The depth profiles of C and O are in units of counts/s and labelled in the graphs.

### Capacitance measurements and NanoESCA analysis

Capacitance measurements were performed on uncoated ZIRLO tubes before and after hot steam exposure (400 °C for 4 days) and on 300 nm NCD-coated ZIRLO tubes exposed to the same hot steam conditions. The types and densities of the defects in the surface films were determined (Table S5). Because a balance must be established between the metal and semiconductor, there cannot be any electrochemical potential difference between the materials in contact; i.e., the Fermi levels must coincide between the metal and semiconductor. The nature of the electric field at the ZrO_2_/Zr interface depends on the semiconductor type (donor/acceptor densities) and markedly influences oxygen transport through the oxide/metal interface.

The impedance spectra of samples exposed to hot steam (4 days at 400 °C) (Fig. S4) showed a predominant capacitance contribution from the zirconium oxide corrosion layer over a very broad frequency range, from 10^6^ Hz to 10^0^ Hz. A slightly higher extent of impedance dispersion was found (both the phase angle and the slope of log /Z/ vs. log f were lower) for the zirconium oxide formed on the NCD-coated samples. The impedance spectra were fitted by using an equivalent circuit model (see Methods). The dashed lines denote the linear slope regions used to calculate the densities of defects (additional donors/acceptors). As expected, the surface layers of hot-steam-processed ZIRLO both with and without NCD protective coatings exhibited semiconductive behavior.

The observed impedance dispersion in the high-frequency range showed no signs of the characteristic response of the NCD coating. The impedance response was dominated by the composite and relatively uniform effect of the carbon-enriched oxide (as a result of the diffusion of carbon into the oxide, as found in the SIMS analysis) and by remnants of the NCD coating. This finding indicated a possible change in the effective dielectric constant from a value of 23 (ZrO_2_) to values between 23 and 6–9 (values typical of diamond layers) (Table S5).

**Figure 8:**
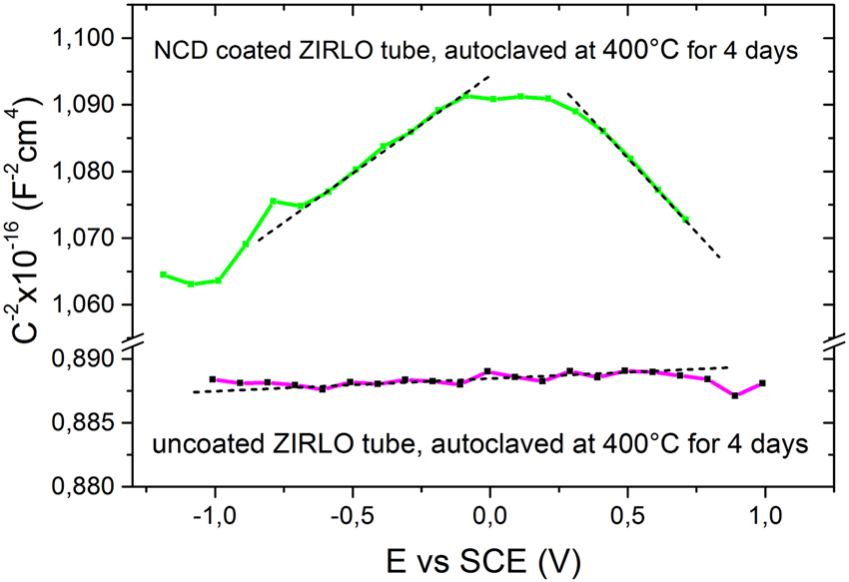
Mott-Schottky plots for NCD-coated and uncoated ZIRLO alloy samples after 4 days in 400 °C steam. The dashed lines denote the linear slope regions used to calculate the densities of defects (additional donors/acceptors). Both the zirconium oxide and the NCD layer clearly exhibited semiconductive behavior.

In Fig. 8, Mott-Schottky plots of uncoated and NCD-coated ZIRLO samples after 4 days of exposure to 400 °C steam are presented. Only positive slopes of the Mott-Schottky plots were recorded in the case of uncoated ZIRLO samples. Therefore, the oxide films on uncoated and hot-steam-treated ZIRLO were found to exhibit solely n-type semiconductive behavior. This result was consistent with observations of pure zirconium^22^ and of Zr-Nb^23^ and Zr-Sn alloys^10–14, 24^. The results of the doping density analysis are presented in Table 2, where N_A_ and N_D_ denote the acceptor and donor densities, respectively. A high donor density (characteristic of an n-type semiconductor) was found for the uncoated ZIRLO both before and after hot steam exposure, whereas the NCD-coated ZIRLO samples after hot steam exposure showed both p-type (acceptor-containing) and n-type semiconductive behavior (Table 2).

**Table 2:** Acceptor and donor densities obtained from the Mott-Schottky plots for uncoated ZIRLO before and after hot steam exposure and for NCD-coated ZIRLO samples after hot steam exposure.

Sample	N_A_ [cm^−3^]	N_D_ [cm^−3^]
ZIRLO	—	4 × 10^20^
ZIRLO, 4 days at 400 °C	—	2.3 × 10^18^
ZIRLO with 300 nm NCD, 4 days at 400 °C	4.4 × 10^16^	2–3 × 10^16^

The uncoated ZIRLO exhibited n-type semiconductive behavior, whereas the NCD-protected ZIRLO after hot steam exposure exhibited mixed p- and n-type semiconductive behavior.

The work function values obtained with the NanoESCA system support the results of the capacitance measurements. Typical Ultraviolet Photoemission Spectroscopy (UPS) spectra used for work function determination are presented in Fig. S5. Compared with standard ZrO_2_^28–30^, uncoated ZIRLO samples exposed to hot steam for 4 days at 400 °C were found to have a relatively low ZrO_2_ work function of 2.0 eV, thus indicating an elevated position of the Fermi level. The Fermi level moves up in the presence of donors, and a high level of donors was indeed detected (Table 2). By contrast, for NCD-coated ZIRLO samples exposed to the same conditions, we observed a higher value of the surface ZrO_2_ work function, 4.0 eV (Fig. S5). This result suggested the presence of acceptors, thereby implying p-type semiconductivity, i.e., a position of the Fermi level that is closer to the valence band. The results presented in Table 2 are well consistent with such an interpretation.

## Discussion

In this work, a new anticorrosion strategy for Zr nuclear fuel cladding tubes and plates was presented and explained. Protective NCD layers were deposited on the surfaces of ZIRLO nuclear fuel cladding samples and ZIRLO plates by using an MW-LA-PECVD apparatus. A wide range of tests and analyses demonstrated the ability of such NCD layers to protect ZIRLO against corrosion in water-cooled nuclear reactor environments.

These NCD layers were shown to be stable and to exhibit strong adhesion to ZIRLO surfaces. Because of NCD’s known high Young’s modulus^27^, NCD protective layers were able to withstand volume expansion during heating without any fundamental impairment of their integrity (as shown via SEM imaging and Raman spectroscopy).

After long-term processing (for up to 195 days) in 360 °C hot water (in accordance with ASTM standard procedures)^21^, a larger relative weight gain was found for ZIRLO samples with unprotected surfaces than for NCD-coated samples. The relative weight gain of the NCD-coated ZIRLO samples was decreased by 35–55%. This decrease was attributed to the restricted oxidation and hydrogen uptake of the NCD-protected samples^12^. NCD layers were also found to protect ZIRLO surfaces against H uptake: the average hydrogen concentration was markedly greater for uncoated samples. For high-temperature hot steam processing (1100 °C, 1200 °C), the uptake of hydrogen into the unprotected material was an order of magnitude higher than that indicated by the hydrogen concentrations in the NCD-protected samples.

We also confirmed that, after hot steam processing, the surface ZrO_2_ layers of NCD-coated ZIRLO tubes were thinner than the corresponding ZrO_2_ layers in unprotected ZIRLO tubes subjected to the same treatment (as demonstrated via optical microscopy, SIMS, SEM, and capacitance measurements).

Thicker (700 nm and 500 nm) NCD layers provided stronger protection of ZIRLO plates and tubes against oxidation than thinner (300 nm) NCD layers. The best protective effect was achieved by 700 nm NCD coatings on ZIRLO plates: the surface oxidation of these samples after hot water processing (360 °C, from 20 days to 40 days) was decreased by more than 50%. The higher protective efficacy achieved by the NCD coatings for ZIRLO plates compared with ZIRLO tubes was attributed to the inner surfaces of the tubes not being fully covered with NCD, whereas in the case of ZIRLO plates mounted vertically in the deposition chamber, all surfaces were covered with homogeneous NCD layers^15^. Notably, the MW-LA-PECVD apparatus, if scaled up as necessary, would be capable of homogeneously coating the full lengths of nuclear rods.

Both the native oxide film on ZIRLO and the oxide formed after 4 days of exposure to 400 °C hot steam exhibited n-type semiconductivity in an approximately 2 μm thick layer. In contrast to unprotected ZIRLO tubes, NCD-protected ZIRLO samples exposed to the same conditions (400 °C hot steam) formed an oxide layer that was thinner by 0.4 μm and showed mixed p- and n-type semiconductivity (capacitance measurements and nanoESCA). This effect was correlated with the penetration of carbon into the ZrO_2_ layer (XPS and SIMS).

We tested the ability of NCD layers to protect the Zr alloy against corrosion over a wide range of environmental parameters: temperatures from 360 °C (hot water, ASTM standard procedures)^21^ to 1200 °C (hot steam) and times ranging from 20 minutes in 1200 °C hot steam up to 195 days in 360 °C hot water. The tested thicknesses of the NCD layers were 300, 500 and 700 nm, and the NCD layers were applied to both ZIRLO plates and ZIRLO tubes. However, the main contribution of this work lies in providing a detailed explanation of the anticorrosive mechanism of NCD. The surface-protective materials that have previously been tested for use on Zr alloys, have been more or less unsuccessful^5–7^, and typically homogeneous. By contrast, the anticorrosive effect of NCD, which is a heterogeneous material (containing both graphite and diamond), was found to be very significant, and, notably, an NCD layer protects ZIRLO against corrosion in a unique way. In this work, we demonstrated and explained the complex mechanism of the surface protection provided by an NCD layer on ZIRLO against H and O uptake. This protection occurs at three basic levels: 1. the NCD protects the ZIRLO surface from directly interacting with water molecules; 2. carbon atoms penetrate into the ZIRLO from the NCD, thus creating carbides and becoming incorporated into ZrO_2_, thereby making conditions less favorable for subsequent O and H uptake; and 3. the carbon from the NCD changes the electrical properties of the ZrO_2_ and creates less favorable conditions for Zr oxidation at the ZrO_2_/Zr interface. These aspects of the protective mechanism are discussed in greater detail below:

**Aspect 1**, the stable NCD layer prevents the Zr alloy from directly interacting with water. The composition of NCD is not homogeneous - the NCD layers at the ZIRLO/NCD interfaces contained relatively large amounts of sp^2^-hybridized C, whereas the surfaces of all NCD layers were formed almost exclusively of hard and inert diamond^17^. The NCD layer at ZIRLO/NCD interfaces, where the ratio of grain boundaries to grains is higher, consists of a higher level of sp^2^-hybridized C. Mainly from sp^2^-hybridized C at the ZIRLO/NCD interface C penetrates into the underlying layers. On the contrary, the surface of NCD, where crystalline grains are fully formed, consists of practically only hard and inert diamond^17^ and therefore forms a strong barrier between the water and ZIRLO. Because of its composition and the conditions of its deposition, such an NCD layer is able to withstand volume changes due to thermal expansion and therefore does not suffer from delamination^15–17^.

**Aspect 2**, during NCD growth and hot steam processing, carbon from the NCD layer penetrates into the underlying material, thus creating carbides, mainly in the zirconium oxide layer and also at the ZrO_2_/Zr interface (Fig. S6). In this manner, carbon changes the original material such that conditions become less favorable for the penetration of oxygen and hydrogen through the ZIRLO surface layer.

**Aspect 3**, the penetrating carbon causes the n-type semiconductivity of the original ZrO_2_ layer to change to a mixed n- and p-type semiconductivity, which also affects the Zr oxidation conditions. Under the typical conditions of hot steam nuclear reactors, the Zr oxide film grows with a large number of defects that cause the ZrO_2_ to exhibit n-type semiconductivity^11–14, 22^. In the contact region between the metal and the semiconductor, a potential difference arises because of the redistribution of charges (Figs S6 and S7). The electric field thus established can markedly affect the transport of charge carriers through the ZrO_2_/metal interface. The presence of impurity atoms in the oxide region affects not only the type of conductivity - electron or hole - but also the difference in chemical potentials and the electric field distribution near the contact region with the oxide film^11, 12^. For unprotected ZIRLO with an n-type semiconductive surface layer, O anions originating from the dissociation of water (2O^2−^ + 4 H^+^ → 2H_2_O) pass redundant electron(s) through the n-type semiconductive ZrO_2_/Zr alloy interface, thus enabling these oxygens to directly interact with/oxidize the zirconium (Figs S6 and S7)^11–13^. This process occurs alongside a process in which Zr atoms lose electrons at the ZrO_2_/Zr interface and subsequently interact with O^2−^ anions, thus also producing ZrO_2_^10, 13^. The electric field established at the semiconductor/metal interface (Fig. S7) depends on the type of semiconductivity (n or p); that is, the type of semiconductivity defines the nature of the possible charge transport through the ZrO_2_/Zr interface and results in different conditions for Zr oxidation^10, 11, 24^. The mixed n- and p-type semiconductivity of the ZrO_2_ that forms in NCD-protected ZIRLO changes the charge distribution, thereby decreasing the electric field in the ZrO_2_ layer and making conditions less favorable for Zr oxidation.

Our results have substantial relevance for various applications. At high temperatures (1100 °C), the investigated NCD layers may fully serve as passive elements for nuclear safety. Also very important is these layers’ effective protection of Zr alloys against oxidation and hydration under standard operating conditions (360 °C). The corrosion of Zr alloys degrades their ability to satisfy criteria for fuel cladding operability, thus leading to the need to replace rods containing nuclear fuel, owing to Zr alloy surface degradation. Better protection of fuel rod surfaces against corrosion would allow for the prolongation of nuclear fuel usage. We found that the oxidation of NCD-coated ZIRLO surfaces after more than 100 days in 360 °C hot water was significantly decreased (35–55%) compared with that of unprotected ZIRLO processed under the same conditions. The NCD-protected ZIRLO alloys also exhibited lower hydrogen concentrations than the unprotected samples under all investigated conditions; in particular, for high-temperature steam exposure (at 1100 *°*C for 1 hour, i.e., accident conditions), the NCD-protected ZIRLO samples showed much lower hydrogen uptake than unprotected ZIRLO samples.

Radiation tolerance of NCD layers grown in the MW-LA-PECVD apparatus as important parameter for application in nuclear reactors was simulated and published in our previous work^16^. We have studied^16^ resistivity of NCD layer as a set of nanodiamond grains and sp^2^ hybridized C against radiation damage. The behavior of NCD coated Zr alloy in a neutron flux environment was simulated by Fe ion beam irradiation of the complex material (Fe^2+^, 3 MeV, room temperature, fluency of 1.95 × 10^16^ cm^−2^). Heavy ions bombardments have been widely used to simulate neutron damage of various materials^36^ and in our specific case no significant changes in structural integrity of NCD layer were found^16^. It should be noted that radiation damage stongly depends on radiation dose and also on specific material setting (i.e. monocrystalline or polycrystalline diamond, sp^2^ C phase)^16, 19, 20^.

It should be emphasized that NCD layer prevents the Zr alloy from directly interacting with water environment. Beside that, carbon from NCD layer penetrates into the underlying material and changes its properties, such that uptake of oxygen and hydrogen into Zr material is significantly decreased. Protective NCD layers can prolong the lifetime of nuclear cladding and, consequently, enhance nuclear fuel burnup. The combination of NCD coatings with other materials may be a very promising step toward achieving the effective protection of Zr alloys against corrosion under exposure to hot steam or hot water. According to tests conducted at the Halden Material Testing Reactor (Norway), NCD-coated ZIRLO claddings with properly grown NCD films are a possible candidate for Accident Tolerant Fuel in commercially operated reactors.

## Experimental methods

### Growth of NCD

ZIRLO fuel cladding tubes (25 mm in length and 10 mm in diameter) and ZIRLO plates (20 mm × 20 mm × 0.1 mm) were immersed (covering the internal and external surfaces of the tubes and both sides of the plates) in a colloidal solution of diamond nanoparticles (NanoAmando). The diamond nanoparticles acted as seeds and therefore as nucleation sites for NCD layer growth. Each ZIRLO sample was then coated with a homogeneous NCD layer grown using an MW-LA-PECVD apparatus (Fig. S1) under the following conditions: a gas mixture of H_2_ + CH_4_ + CO_2_, a process pressure of <1 mbar, a microwave power of 2 × 3 kW, and a temperature of approximately 600 °C. Because of the diffuse nature of plasma at low process pressures, the growth of NCD layers was achieved over the entire outer circumference of ZIRLO tubes mounted horizontally in the deposition chamber. However, the inner surfaces were only partially coated. This lack of internal coverage is further discussed above with respect to the interpretation of the results. In the case of ZIRLO plates mounted vertically in the deposition chamber, both surfaces were covered with NCD layers. NCD layers of 200–300 nm (labeled as 300 nm), 400–500 nm (labeled as 500 nm) and 600–700 nm (labeled as 700 nm) were produced. Optical microscopy, Raman spectroscopy and SEM were used to confirm the NCD layer coverage of the ZIRLO. Notably, the MW-LA-PECVD apparatus could be scaled up such that it would be capable of coating the full lengths of nuclear rods.

It should be emphasized that the coverage of the inner portions of the tubes can be avoided by sealing both tube ends before the NCD layer deposition. The NCD layer then covers only the outer surface of the tube. NCD coated tube samples for Halden Material Testing Reactor (Norway) were made in this way//coated just on the outer surface.

### Sample exposure to nuclear reactor environments under normal and accident conditions

To simulate the long-term protective capabilities of NCD layers under normal conditions in a nuclear reactor, NCD-coated and reference uncoated ZIRLO samples were subjected to a series of high-temperature autoclave water tests. In accordance with ASTM standard procedures^21^. the samples were exposed for 6, 15, 20, 30, 40, 90, 120, 150, 170 and 195 days in hot water (360 °C) at a pressure of 16 MPa, close to the conditions found in the primary circuit of a PWR, at the Westinghouse facilities in Pittsburgh, USA. No additional chemicals were used in the autoclave water.

To simulate the protective capabilities of NCD layers against hot steam under normal and accident conditions in a nuclear reactor, NCD-coated and reference uncoated ZIRLO samples were subjected to a series of high-temperature steam tests. The samples (tubes or plates) were placed in a silica glass tube reactor, which was placed inside a furnace. The samples were heated to the required temperature and for the required duration (400 °C/4 days at 15 MPa and less than 10 ppb of oxygen, 900 °C/1 h, 1100 °C/1 h and 1200 °C/20 min) in an inert argon atmosphere at atmospheric pressure. All steam exposures were performed isothermally. After hot steam processing, the steam/water flow was switched off after the required time, and the sample was slowly cooled down under an argon atmosphere.

### XPS analysis

XPS measurements were performed using an ESCA Probe P (Omicron Nanotechnology) with a primary X-ray source of monochromatized radiation from an Al anode (1486.7 eV). The constant analyzer energy (CAE) mode was used, at pass energies of 50 eV for overview spectra and 30 or 20 eV for detailed spectra. Charge compensation was achieved by using an electron gun at very low electron energies (between 1 eV and 2.5 eV) to protect the sample. Measurements of copper and calibration constants derived from these spectra were used for intensity calibration. The full widths at half maximum of the components used for the analysis of line details were based on experimental experience with the spectra of carbon and silver and were consistent with the capabilities of the instruments used. Spectral evaluation was conducted using CASA XPS software; the area of the peaks after calibration and a database of relative sensitivity factors were used for the determination of elemental concentrations. Chemical species were identified using XPS databanks. An ion gun (ISE5) connected to the preparation chamber was operated with argon ions, typically at an energy of 5 keV.

### Mechanical and tribological measurements

The analysis of the mechanical and tribological properties of the samples was performed using a fully calibrated NanoTest instrument equipped with diamond indenters at room temperature, operating in load-controlled mode. A three-sided Berkovich indenter was used in nanoindentation experiments to evaluate the indentation hardness, elastic modulus and creep. Nanoindentation was performed at a peak load of 1 mN, with both the loading and unloading rates set to 0.5 mN/s. This relatively low load ensured, in the case of the NCD films, a maximum penetration depth of approximately 10% of the layer thickness to ensure that the calculated values of the mechanical properties could be regarded as layer dominated. Standard analysis procedures were used to calculate the indentation hardness and the decreased elastic modulus from at least 10 independent measurements for each sample^33^. Creep experiments with a dwell period of 300 s at a load of 200 mN were performed to explore the composite response of the film/ZIRLO system to constant loading. To quantify the deformation tendencies of the NCD layers and the ZIRLO samples before and after hot water/steam exposure under a constant indentation load, the parameter P introduced by Goodall and Clyne was used^31^. This parameter is defined as the product of the slope of the steady-state part of the creep curve, in our case 150–300 s, and the increase in depth at the end of the constant-load period. For a higher value of P, higher deformation is expected to occur during constant-load creep^32^.

The scratch resistance of the uncoated and NCD-coated ZIRLO samples was tested using a sphero-conical Rockwell indenter with a nominal radius of 10 µm. Progressive scratch tests up to a maximum load of 500 mN were performed in a 3-step procedure consisting of initial topography measurements followed by scratch and final topography measurements. The topography measurements were performed over the entire scratch length at a low load of 0.02 mN to avoid wear. During the scratch procedure, the applied topographic load was initially constant over the first 50 μm and then ramped to the maximum load at a constant loading rate of 13.8 mN/s. All scans were performed at a scan speed of 10 μm/s over a total scan length of 450 μm. Evaluation of the scratch tests was performed on the basis of the indenter on-load and depth records and analysis of the residual scratch tracks using a confocal laser scanning microscope (Olympus LEXT 3000).

### Thermogravimetry, mass spectrometry and optical microscopy of the metallographic cross sections of samples

Thermogravimetry measurements were performed at elevated temperatures between 900 and 1100 °C. The samples were placed on an alumina holder in a NETZSCH thermobalance (model STA 409). Argon was used as an inert cover gas. Steam exposures were performed isothermally, without any changes in the steam or cover gas flow rates. All high-temperature oxidations were performed under an argon flow rate of 3 l/h and a steam flow rate of 3 g/h. The overall weight gain (w_g_), which is a commonly used quantity to describe the corrosion of a zirconium alloy in a water-steam environment, was determined by measuring the sample’s weight before and after oxidation. This weight was normalized with respect to the total exposed surface area of the sample to compensate for different sample sizes. The units used (mg/dm^2^) are commonly used in engineering practice and calculations. The sample surface area was calculated from the sample dimensions (length and inner and outer diameters) measured before steam exposure. The equivalent cladding reacted (ECR) (in %) for two-sided oxidation was calculated using the following formula^14^:

1 $$ECR=\frac{87.8\,{w}_{g}}{s}$$

where *s* is the thickness of the sample and *w*_*g*_ is the weight gain over the entire surface area.

Off-gas analysis was performed with a Balzers GAM 300 quadrupole mass spectrometer. The hydrogen flow rate was calculated using the measured concentrations of argon and hydrogen, and the argon flow rate was measured with a volumetric flowmeter. Overall hydrogen production was calculated as the integral of the hydrogen flow rate. Hydrogen production was normalized to the sample surface area to compensate for different sample sizes.

Cross sections were acquired from the central parts of samples processed under 1000 °C hot steam by using an optical microscope. The samples were hot pressed into electrically conductive transparent resin (Bakelite), which is suitable for sample analyses via optical microscopy. The samples were ground using an automatic polishing device with a polishing head with individual contact pressure. The contact pressures were chosen according to the temperature at which each sample was exposed. Polishing was performed by using the same polishing device with an individual contact pressure of 35 N for all types of exposures. A classic canvas and a colloidal mixture of SiC (Colloidal Silica Polishing Suspension) from MasterMet were used for polishing. The samples were observed using an optical microscope after each polish.

### SEM

The surface morphologies of the NCD layers were analyzed by using a Tescan FERA 3 scanning electron microscope. To minimize the interaction volume during imaging, the accelerating voltage in high-resolution mode was kept in the range of 2–5 kV. EDS composition analysis was performed using an EDAX Octane Super 60 mm^2^ detector with an acceleration voltage of 5 kV for elemental analysis. The structures were opened using a Xe-FIB.

### Raman spectroscopy

To determine the NCD layer composition (sp^2^ and sp^3^-hybridized carbon), the deposited layers were characterized with Raman spectroscopy performed at room temperature using a Renishaw InVia Raman Microscope under the following conditions: a laser excitation wavelength of 488 nm (25 mW), ×50 Olympus objective, 65 µm slits, spot focus, and a grating of 2400 lines/mm. Spectra were acquired at various points across the samples to probe the homogeneity of the deposited NCD layers.

### SIMS

SIMS measurements were performed in depth profiling mode with a Cameca IMS 7 f magnetic sector instrument. A Cs^+^ primary ion beam with an impact energy of 15 keV, a current of 20 nA and an impact angle of ~23° from the surface normal was raster scanned over an area of 100 μm × 100 μm, thus resulting in a sputter rate of ~1.4 nm/s. The depth scale of the SIMS craters was calibrated using a stylus profilometer. The instrument was operated at a low mass resolving power of M/ΔM ~400. Standard precautions were taken to limit the distortions of the SIMS depth profile data caused by crater edge effects. Secondary ions were detected in single-ion counting mode using an electron multiplier or, for high count rates, using a Faraday cup and an electrometer amplifier. An electron beam in self-compensation mode was used for charge compensation during the analysis. The vacuum pressure in the analysis chamber during the measurement was approximately 1 × 10^−9^ mbar. Negatively charged secondary ions were monitored.

### Hydrogen concentration measurements

The amount of hydrogen dissolved in the ZIRLO after steam exposure (at either a low temperature simulating operating conditions or a high temperature simulating accident conditions) was determined with a G8 GALILEO analyzer. This analyzer operates on the basis of the inert gas fusion principle, which requires the fusion of the sample material in a graphite crucible at high temperatures. The concentration of the outgoing hydrogen is subsequently measured with a mass spectrometer. No correction for an oxide layer was applied; consequently, for heavily oxidized samples from thermogravimetric experiments, the measured value of the hydrogen concentration does not exactly reflect the actual concentration in the sample.

### Capacitance measurements

Impedance spectra and the dependence of capacitance on potential (Mott-Schottky plots) were measured in a 3-electrode cell at room temperature in 0,5 M K_2_SO_4_ and a borate buffer (pH = 9,2), respectively. A saturated calomel electrode was used as the reference electrode, and the NCD-coated and uncoated ZIRLO tubes and plates before and after oxidation for 4 days in 400 °C steam were used as the working electrodes. A platinum mesh coaxial electrode served as the counter electrode. Included in the measurement setup was a Reference 600 potentiostat (Gamry). Impedance data were measured after the stabilization of the open-circuit potential for 1 h. The impedance spectra were measured in the frequency range from 10^6^ Hz to 10^−2^ or 10^−3^ Hz, using a 5 mV perturbation signal. Polarization was applied in successive steps of 100 mV in the anodic direction over the potential range of −1 V to +1 V. The amplitude of the perturbation signal was 5 mV, and the frequency was 1 kHz. Under the assumption of a serial connection between the solution resistance and electrode capacitance at high frequencies, the capacitance was calculated from the relation:

2 $$C={(-{Z}_{i}2\pi f)}^{-1}$$

where Z_i_ is the imaginary part of the impedance, and f is the frequency. The impedance spectra were fitted by using the equivalent circuit model depicted in Fig. S8. An RC* parallel combination was used to express the response of a surface layer (NCD and/or oxide) and an Rf - Cdl parallel combination in series to RC*, describing faradaic resistance and double- layer capacitance effects.

In the RC* term, R represents the layer resistance, and C* is the complex capacitance term introduced by Jonscher^37^ to describe the impedance dispersion induced by a non-ideal dielectric response. The impedance of a Jonscher element can be expressed as follows:

3 $${C}^{\ast }={C}_{\inf }+\frac{1}{Q{(j\omega )}^{n}}$$

where C* is a complex capacitance; Q is a constant related to the dielectric dispersion; the exponent n is the dispersion index, which represents the extent of capacitance dispersion (for an ideal capacitance, n = 1); j is the imaginary unit; and ω is the angular frequency. C_inf_ is defined as the capacitance value at infinite frequency. C_inf_ is independent of frequency and can be directly related to the dielectric layer thickness by the following expression for a flat capacitor:

4 $$\delta =\frac{A{\varepsilon }_{r}{\varepsilon }_{0}}{{C}_{\inf }}$$

where *ε*_*0*_ is the permittivity of vacuum (8.854 × 10^−14^ F/cm), *ε*_*r*_ is the relative permittivity of the layer, and *A* is the area. In a Mott-Schottky analysis, the impedance of the electrode-electrolyte interface is measured at a high frequency as a function of the imposed potential. The capacitance values are obtained from the imaginary part of the impedance:

5 $$C=-{(\omega Z^{\prime\prime} )}^{-1}$$

The interfacial capacitance *C* measured at high frequency can be described as a series combination of a capacitive contribution from the surface layers, *C*_*surf*_, which includes the oxide- and NCD-layer responses; a contribution from the space charge region developed in the defective barrier layers in the NCD layer and/or the oxide layer, *C*_*sc*_^22^; and a contribution from the Helmholtz layer at the film-electrolyte interface, *C*_*H*_:

6 $$\frac{1}{C}=\frac{1}{{C}_{surf}}+\frac{1}{{C}_{SC}}+\frac{1}{{C}_{H}}$$

In the case of high-frequency measurements, the Helmholtz capacitance term can be neglected (C_H_ >> C_ox_, C_sc_), and the dielectric response term is independent of the potential. The relationship between the measured capacitance and the potential can be expressed using Mott-Schottky theory for the case of n-type conductivity as follows:

7 $$\frac{1}{{C}^{2}}=\frac{1}{{C}_{surf}^{2}}+\frac{2}{{\varepsilon }_{r}{\varepsilon }_{0}q{N}_{D}}(V-{V}_{fb}-\frac{kT}{q})$$

Here, *ε*_*r*_ is the dielectric constant of the surface film (in this study *ε*_*r*_ = 23 was used for an oxide film and *ε*_*r*_ = 10^−17^ was used for an NCD-coated sample after a 400 °C hot steam oxidation test), *ε*_*0*_ is the permittivity of vacuum, N_D_ is the donor density (cm^−3^), *q* is the elementary charge, *V* is the applied potential, *V*_*fb*_ is the flat-band potential, *k* is the Boltzmann constant, and *T* is the absolute temperature.

The bulk surface film capacitance term is independent of voltage and can cause a shift in the flat-band potential value (the intercept of the linear part of the Mott-Schottky plot when extrapolated to zero capacitance). It does not influence the slope of the linear part of the Mott-Schottky plot, from which the donor density is determined^23^.

### XPS, UPS and work function analysis with a NanoESCA system

Uncoated and NCD-coated ZIRLO samples after hot steam tests were subjected to analyses of their work functions and surface potentials. For this purpose, a NanoESCA (Oxford Instruments Omicron Nanoscience) photoemission spectrometer based on a Photoelectron Emission Microscopy (PEEM) column and a double hemispherical imaging energy filter was used. This apparatus enables XPS, UPS and PEEM measurements and analyses using various excitation sources: monochromatic Al Kα_1,2_ X-rays, an He discharge lamp (FOCUS HIS 13) and an Hg lamp. The valence band spectra for the determination of the Fermi level and the valence band maximum were acquired via XPS. Work function mapping was performed via PEEM using an Hg lamp. Furthermore, the work function values were also probed by obtaining UPS spectra. The work function of each sample was extracted from the low-kinetic-energy cut-off of the UPS spectrum. The samples were briefly Ar sputtered to remove surface contamination.

## Electronic supplementary material

Supplementary information
