# Supplementary material for: Nanocrystalline diamond protects Zr cladding surface against oxygen and hydrogen uptake: Nuclear fuel durability enhancement
Source: Sci Rep. 2017 Jul 25;7:6469. doi: 10.1038/s41598-017-06923-4 (PMC5526891; doi:10.1038/s41598-017-06923-4)
Supplement: Supplementary file 1 — Supplementary information [file 41598_2017_6923_MOESM1_ESM.pdf]

Nanocrystalline diamond protects Zr cladding surface against oxygen and hydrogen uptake: Nuclear fuel durability enhancement. Supplementary information.

Jan Škarohlíd[1], Peter Ashcheulov[2], Radek Škoda[1], Andrew Taylor[2], Radim Čtvrtlík[3], Jan Tomáštík[3], František Fendrych[2], Jaromír Kopeček[2], Vladimír Cháb[2], Stanislav Cichoň[2], Petr Sajdl[4], Jan Macák[4], Xu Peng[5], Jonna M. Partezana[6], Jan Lorinčík[7], Jana Prehradná[1], Martin Steinbrück[8] and Irena Kratochvílová[2*]

[1]Czech Technical University in Prague, Faculty of Mechanical Engineering, Technická 4 , Prague 6, CZ-160 07, Czech Republic
[2]Institute of Physics of the Czech Academy of Sciences, Na Slovance 2, CZ-182 21, Prague 8, Czech Republic
[3]RCPTM, Joint Laboratory of Optics of Palacký University in Olomouc and Institute of Physics of the Czech Academy of Sciences, 17. listopadu 12, CZ-771 46 Olomouc, Czech Republic
[4]University of Chemistry and Technology, Power Engineering Department, Technická 3, Prague 6, CZ-166 28, Czech Republic
[5]Nuclear Fuel Division, Westinghouse Electric Company, 5801 Bluff Road, Hopkins, SC 29209, USA
[6]Westinghouse Churchill Site1332 Beulah Rd. Pittsburgh, PA 15235 USA
[7]Research Centre Řež, Hlavní 130, CZ-250 68 Husinec-Řež, Czech Republic
[8]Institute for Applied Materials (IAM), Karlsruhe Institute of Technology, Hermann-von-Helmholtz-Platz 1, 76344 Eggenstein-Leopoldshafen, Germany

[*]corresponding author: krat@fzu.cz, phone +420266052524

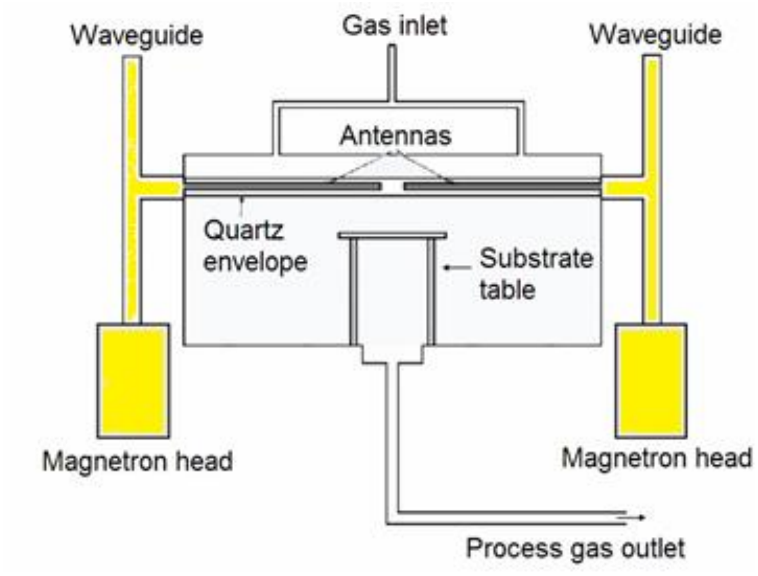

Fig. S1: Schematic of linear antenna microwave plasma enhanced chemical vapor deposition (MW-LA-PECVD) apparatus. A large and diffuse plasma is formed along the quartz envelopes beneath which large 3D objects can be coated.

Mechanical and tribological measurements

The nanoindentation experiments including indentation creep test confirmed the high mechanical durability of the NCD coatings typical for such class of material. Indentation hardness and reduced modulus reaches value of (18.5±2.2) GPa and (202±19) GPa, respectively. These values are comparable with those of a-SiC coatings also considered as a possible cladding protection material.

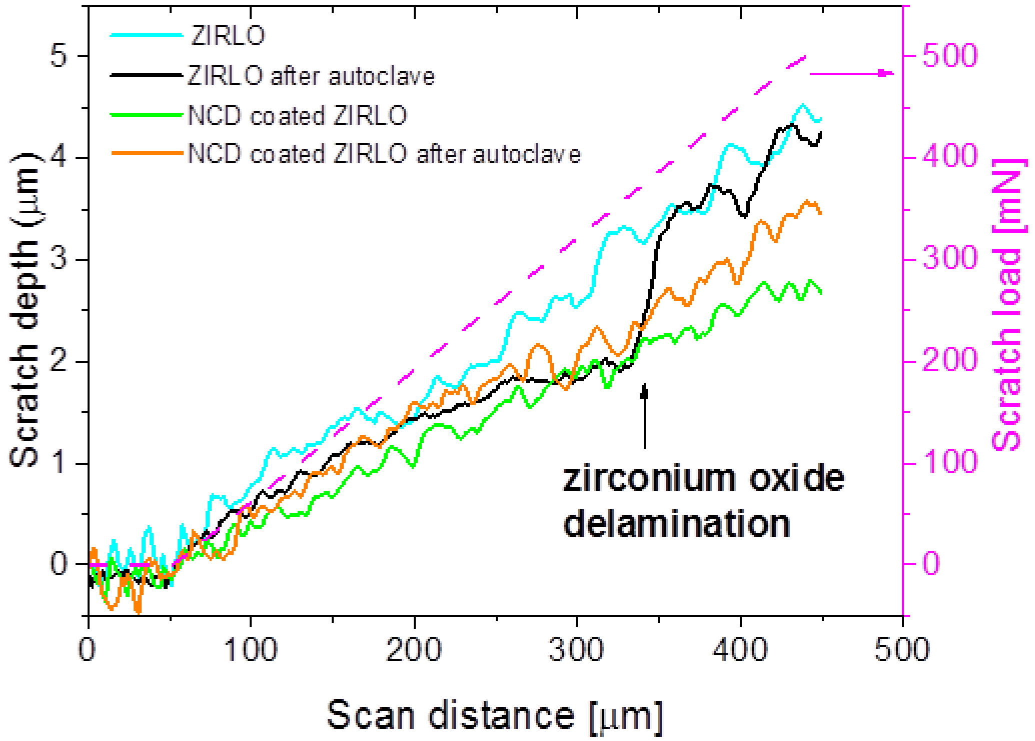

Fig. S2: Scratch test depths profiles for uncoated and NCD coated ZIRLO before and after hot steam test (4 days at 400 °C); dash line represents scratch load. The sudden drop in scratch depth in the case of autoclaved uncoated ZIRLO (scan distance of ~330 µm; critical load of ~370 mN) reflects the potential vulnerability of the bare ZIRLO under conditions of mechanical loading. Although ZrO_2 improves the surface hardness its abrupt failure manifests the unreliable performance under extreme conditions. Conversely, deposition of NCD layer provides an extra level of surface mechanical durability during and after hot steam exposition. The underlying ZIRLO remains protected even under severe scratch loading. The scratch resistivity of NCD protected ZIRLO remains unchanged following 4 days in 400 °C hot steam.

A

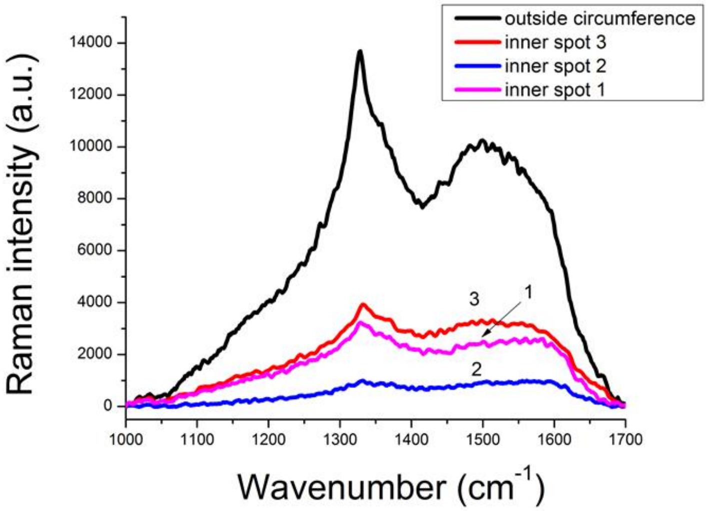

B)

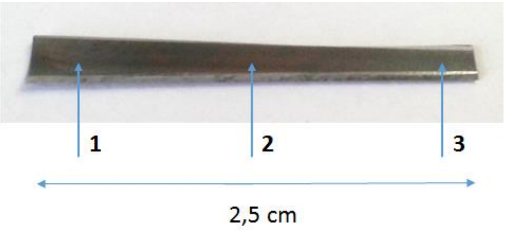

C)

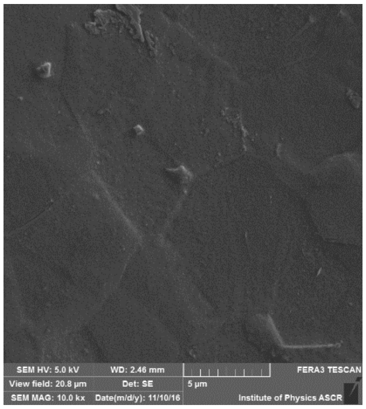

Fig. S3 (a) 488 nm Raman spectra of the NCD layer deposited on the outer surface and a variation of the layer quality on the inner surface – decrease in the intensity of the Raman signal as well as in sp3 phase as the distance from the tube's edge increases; (b) Photo of the specimen cut from the coated ZIRLO tube and corresponding points of the collected Ramanspectra; (c) SEM images of the inner part of ZIRLO tube showing a presence of a coating which do not exhibit clear crystalline structure as the NCD layer on the outer surface.

Table S1.: Average hydrogen concentration measured after hot water processing at 360 °C for 90 days of uncoated and NCD coated (500 nm) ZIRLO samples (tubes and plates). Compared to NCD protected ZIRLO a larger hydrogen concentration in unprotected samples can be observed confirming that NCD layers protect ZIRLO against H penetration.

**Sample**	**Average hydrogen concentration (ppm)**
tube 500 nm NCD	31
plate 500 nm NCD	21
uncoated tube	37

Table S2. Weight gain, equivalent cladding reacted and overall hydrogen production of NCD coated (300 nm) and uncoated ZIRLO™ samples after 60 min. at 900-1100°C in hot steam. Values confirm that even after 60 minutes in 1100°C hot steam the NCD layer protects ZIRLO tubes against oxidation and hydrogen uptake.

**Temperature**	**Weight gain**		**Equivalent cladding reacted**		**Hydrogen produced**	
[°C]	[g.dm^{-2}]		[%]		[l.dm^{-2}]	
	Coated	Reference	Coated	Reference	Coated	Reference
900	0,55	0,64	8,45	9,90	0,56	0,69
1000	2,14	2,87	32,97	44,19	2,20	3,44
1100	3,83	3,98	59,05	61,35	4,69	4,96

Table S3. Average hydrogen concentration measured after hot steam processing at 1100 and 1200 °C hot steam for 60 min and 20 min respectively of uncoated (reference) ZIRLO tubes and NCD coated (500 nm) ZIRLO samples. Compared to NCD protected ZIRLO a much larger hydrogen concentration was found in unprotected samples confirming NCD layer's protection of Zr alloy against H penetration.

Temp. (°C)	Time (min)	Weight gain ($g.dm^{-2}$)		Average hydrogen concentration (ppm)	
		Coated	Reference	Coated	Reference
**1100**	**60**	**2,14**	**2,87**	**51**	**571**
**1200**	**20**	**3,83**	**3,98**	**63**	**520**

Table S4: Oxygen content (measured by EDS) and ZrO_2 layer thickness in uncoated and NCD coated (300 nm) ZIRLO tubes, both after oxidization in hot steam at 400 °C for 4 days. A higher amount of oxygen was detected in the ZrO_2 layer of unprotected samples.

	**ZIRLO autoclaved**	**ZIRLO covered by 300 nm NCD, autoclaved**
Atomic % of oxygen in ZrO_2	33 ± 2	19 ± 1
ZrO_2 thickness (µm)	2.16 ± 0,05	1.7 ± 0,05

Table S5. Capacitance values and dielectric thickness values of reference (i.e. uncoated and no hot steam exposure) ZIRLO and hot steam processed (400°C for 4 days) uncoated and NCD coated (300 nm) ZIRLO tubes. The presented dielectric constants were used to determine the prevailing type of defects in semi conductive layers.

**Sample**	**c_{inf} [$F.cm^{-2}$]**	**ε_r**	**δ [µm]**
Tube - reference	$1.3x10^{-5}$	23	$2x10^{-3}$
Tube 4 days, 400°C hot steam	$1.45x10^{-8}$	23	1.9
Tube 300 nm NCD, 4 days, 400°C hot steam	$1.5x10^{-8}$	10-17	1.5

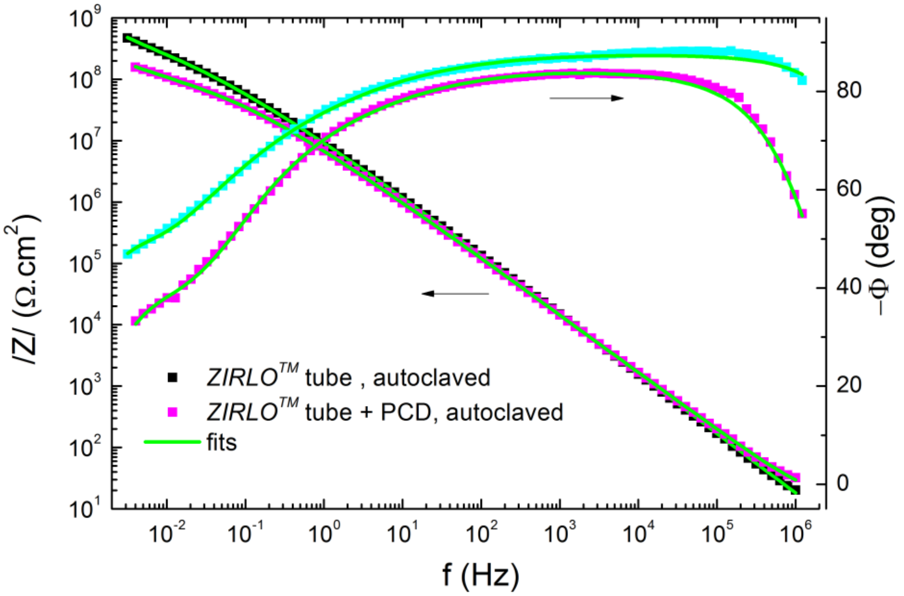

Figure S4. Left: Bode plots of impedance spectra of uncoated ZIRLO Right: Bode plots of impedance spectra of NCD (300 nm) coated and uncoated ZIRLO samples after exposure in steam at 400°C for 4 days. The dominating capacitance contribution from the zirconium oxide corrosion layer in a very broad frequency range 10^6 Hz to 10^0 Hz is clearly seen.

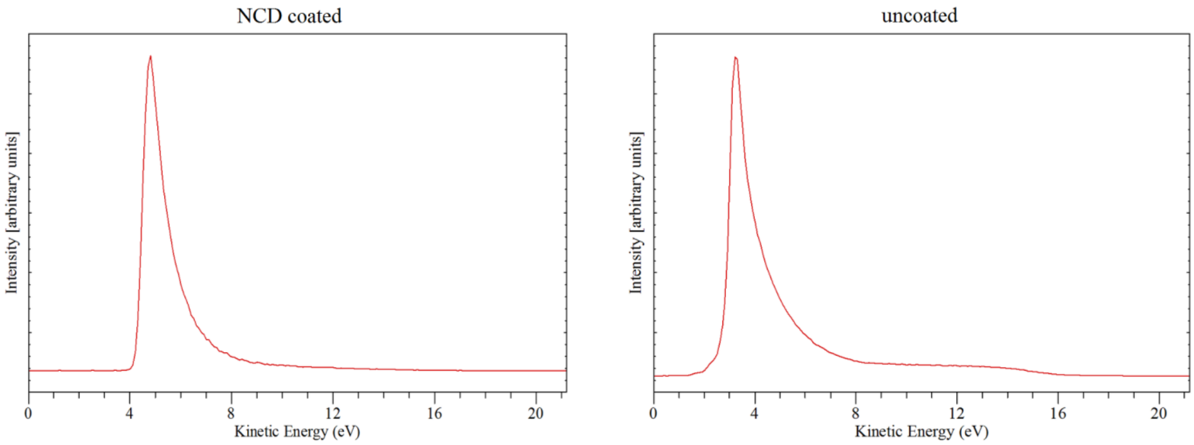

Fig. S5: Typical UPS spectra measured on NCD coated and uncoated ZIRLO alloy samples after 4 days in 400°C steam. A higher value of work function at the NCD coated sample in comparison with the uncoated one is obvious.

A)

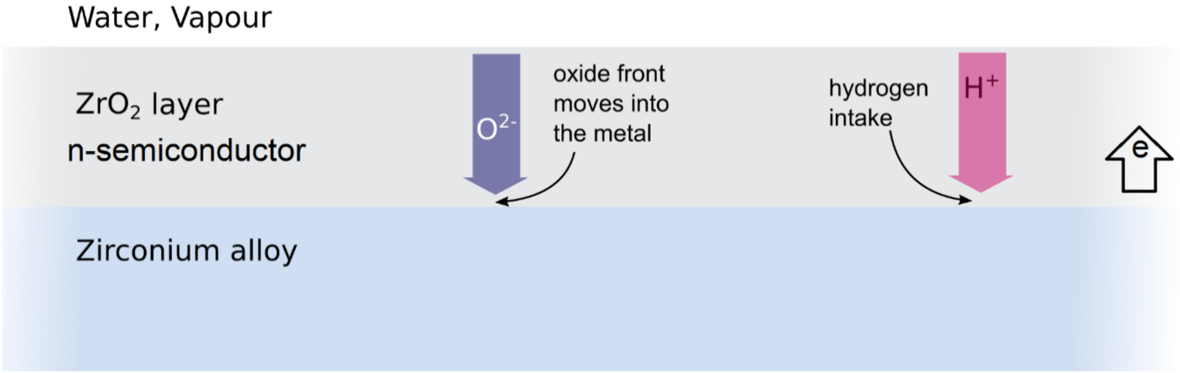

B)

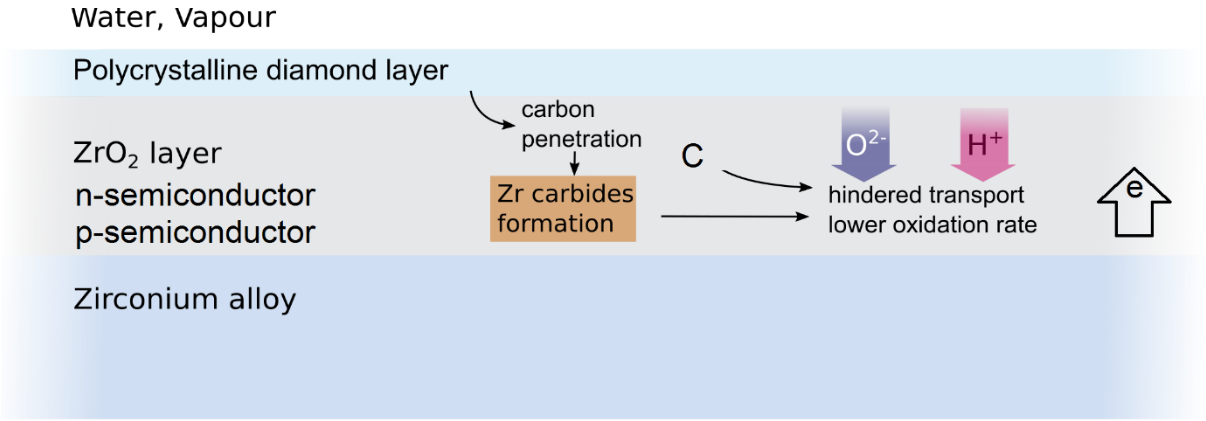

Fig. S6: Schematic showing the transport of H and O from the surface and into the bulk of ZIRLO during hot water/steam exposure for A) uncoated ZIRLO, which is mainly chemically composed of ZrO_2, implying a large uptake of oxygen forming an n-type semiconductor. B) NCD coated ZIRLO, where carbides formed by C penetration from the NCD layer are present in the ZrO_2 layer and the bulk Zr alloy forming a mixture of p-type and n-type semiconductor. The presence of electrical fields and gradients of chemical potentials affect transport mechanisms through the surface layer reducing O and H uptake.

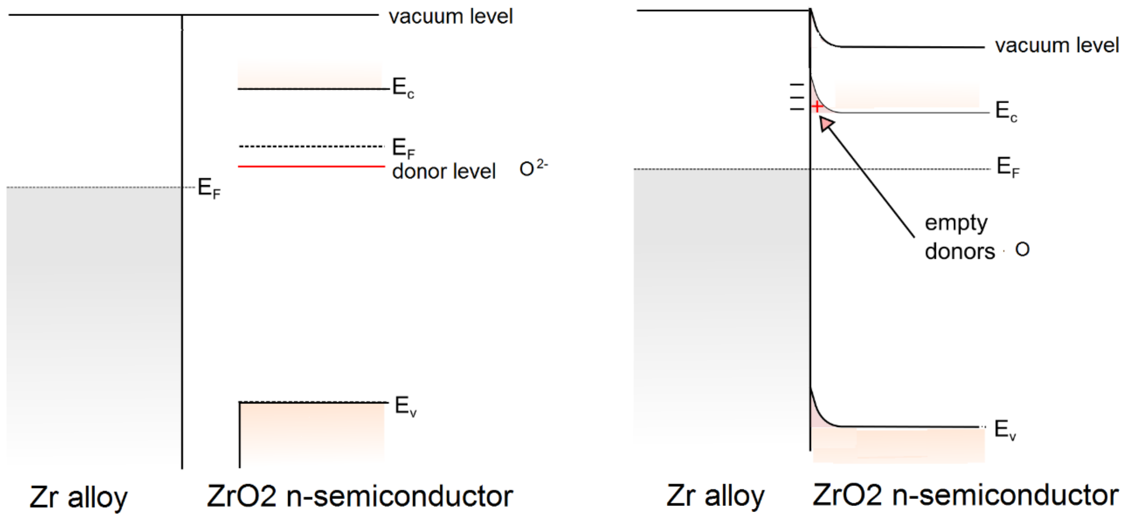

Fig. S7: Band structure of a metal (Zr alloy) and n-type semiconductor (ZrO_2) interface before (left, A) and after metal and semiconductor connection (right). If there is no contact, electrochemical potentials of metal and semiconductor are different then at the first moment after contact diffusion electrical current will flow through the boundary. Flowing will go on until a difference of charges between metal and semiconductor is set-up and an electric field is created so that further current flowing is ceased. Charge is injected through the ZrO_2/Zr interface. E_c denotes conductive band edge, E_v valence band edge and E_f Fermi level. In this work we showed that the oxide film on ZIRLO samples formed during hot steam/water exposure exhibit n-type character. Oxygen anions with an excess of electrons coming from dissociated water molecules increase the concentration of possible electron donors in the n-type semi-conductor. Then at the ZrO_2/Zr interface oxygen anions donate electron(s) through the n-type semi-conductor/metal interface and oxygen finally interacts with Zr to create an oxide.

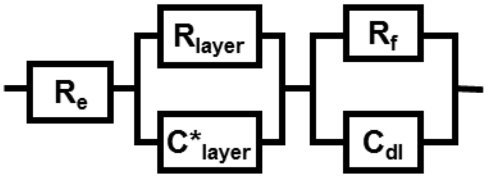

Fig. S8. Equivalent circuit used for fitting of experimental impedance data of uncoated and NCD-coated ZIRLO samples. RC* parallel combination expresses a response of a surface layer (NCD and/or oxide) and R_f -C_{dl} parallel combination, where R_f is faradaic resistance and C_{dl} is double layer capacitance. In the RC* term R has the meaning of layer resistance and C* is the complex capacitance term.
